# High Intensity Focused Ultrasound – Longitudinal Data on Efficacy and Safety

**DOI:** 10.5334/tohm.987

**Published:** 2025-05-05

**Authors:** Betsy Thomas, Gabriele Bellini, Wen-Yu Lee, Yidan Shi, Alon Mogilner, Michael H. Pourfar

**Affiliations:** 1 Department of Neurology, NYU Langone Health, New York, USA; 2 Marlene and Paolo Fresco Institute for Parkinson’s and Movement Disorders, NYU Langone Health, New York, USA; 3 Neurology Unit, Department of Clinical and Experimental Medicine, University of Pisa, Pisa, Italy; 4 Department of Population Health, New York University Grossman School of Medicine, USA; 5 Department of Neurosurgery, NYU Langone Health, New York, USA

**Keywords:** HiFU, Parkinson’s Disease, Essential Tremor, Focused Ultrasound, High Intensity

## Abstract

**Background:**

High intensity focused ultrasound (HiFU) is a relatively new incisionless intervention used for treatment of essential tremor and Parkinson’s disease tremor. Understanding the indications, benefits, risks and limitations of HiFU, as well as how it compares to deep brain stimulation (DBS), is important in guiding appropriate recommendations for prospective patients.

**Methods:**

Current literature on efficacy and safety of HiFU in essential tremor and Parkinson’s disease was reviewed. We additionally reviewed data on the patients who presented to our center for HiFU consultation, including outcomes of patients with low skull density ratios, and distances traveled for the procedure.

**Results/Discussion:**

HiFU is an effective and generally well-tolerated treatment for tremor. Adverse events, especially gait instability, are typically temporary but should be discussed with patients. The risk of tremor recurrence in certain patients with Parkinson’s disease is also of note. Identifying appropriate candidates for either intervention remains crucial and involves considering each patient’s circumstances and preferences, potential adverse effects, and practical aspects like access to follow-up and expectations. Data on bilateral HiFU lesioning, use of HiFU in patients with low skull density ratios, and emerging targets like the pallidothalamic tract are discussed as well.

## Introduction

Despite advances in medical management, medication-refractory tremor, whether related to Parkinson’s disease (PD) or essential tremor (ET), continues to pose a treatment challenge. For over thirty years, the availability of deep brain stimulation (DBS) has helped address this patient population, but many patients were either reluctant or deemed not appropriate candidates for this surgical intervention. More recently, the introduction of high intensity focused ultrasound (HiFU) has opened up a new interventional option which can be weighed against DBS. Being an incisionless procedure, it has proven to be an appealing alternative to a significant number of patients who were wary of open brain surgery, or considered too high a surgical risk because of age or medical co-morbidities. However, a full understanding of the indications, benefits, risks (including both short-term side effects and long-term outcomes) and limitations of both procedures is not always appreciated by either patient or referring physicians. In this article, we will focus on the increasing use of HiFU to treat essential and parkinsonian tremors. Specifically, we will review the current literature as well as our center’s experience guiding patients through the decision-making process, the nature of the procedure and post-procedure management. We will also review how it compares to DBS and discuss the increasingly common factors that lead a patient or center to decide between the two efficacious, approved interventions. Many patients who are reasonable candidates for both treatments have strong opinions about which treatment they would like to pursue, and our discussion would be incomplete without focusing on the factors that influence our patients’ decision-making.

## HiFU: Technical and Procedural Considerations

The preoperative workup for HiFU begins with the history and examination, including an evaluation of the nature of the tremor, characterizing its features and severity and associated disability, often with the assistance of standardized rating scales such as the Tremor Rating Scale (TRS) or Unified Parkinson’s Disease Rating Scale (UPDRS), that can be used to track the patient’s response to interventions. Potential temporary or permanent side effects of the procedure (as will be reviewed below) should be discussed, including the commonly seen side effect of transient worsening of gait and balance after the procedure. Therefore, if the patient is determined to be a good candidate, it is important to discuss this risk and confirm that they have home support, and arrangements made for a walker or wheelchair to use temporarily, if needed.

**Figure 1:**
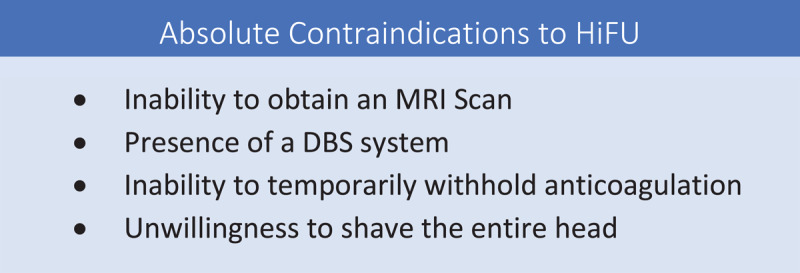
Absolute Contraindications to HiFU.

Absolute contraindications to HiFU are reviewed in Figure 1. As the mean age of HIFU patients is usually older than that of DBS patients, it is not uncommon that patients may be excluded from HIFU due to the presence of an MR-incompatible device. Other absolute contraindications for HIFU include the presence of a DBS system. Although patients with DBS devices can obtain diagnostic MRI scans, they cannot undergo the HIFU procedure. The inability to temporarily withhold anticoagulation is also a contraindication.

At the initial visit, the patient should also be informed that HiFU requires shaving the entire head as a significant number of prospective patients are unaware of this requirement and, in some cases, decline pursuing HIFU as a result (thus making it an important detail that should not be revealed the day of the procedure).

**Figure 2:**
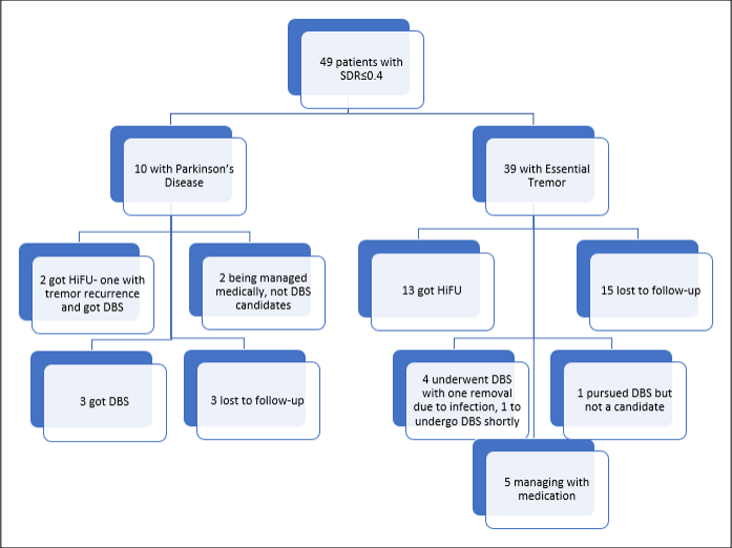
Outcomes of patients with SDR ≤ 0.40.

The next step is determining the patient’s skull density ratio (SDR). The SDR is the ratio between the mean Hounsfield units for marrow and cortical bone [1], determined by performing a specialized CT scan. Thus a higher SDR indicates more homogeneity within the skull and better transmission of ultrasound waves. A lower SDR may negatively impact energy transfer by causing more attenuation and reflection of the waves, potentially leading to a failure to reach the required temperature to create a permanent thalamotomy. The FDA has currently approved HiFU for patients with an SDR of 0.45 ± 0.05 or above. It has been estimated that at least a third of the population has a low SDR [2] and would be excluded from the procedure using this cutoff. However, new studies have demonstrated that HiFU may still be successful in patients with lower SDRs, and, at the time of writing, the FDA labeling has changed to include patients with SDRs as low as 0.30 [3]. At our center, based on our own experience of successfully treating lower SDRs in appropriately selected patients, we have moved away from using 0.40 as a strict cutoff for HIFU, particularly in patients where DBS is absolutely contraindicated, such as advanced age or medical comorbidities. We review with these patients the possibility that effective HIFU may not be possible based on the results of the CT but, in appropriate cases, that it would still allow us to revisit DBS as a potential intervention. Gamma Knife thalamotomy, though not covered in this article, is another non-invasive alternative for patients whose skull density precludes HIFU. To date, our center has seen 221 patients for HiFU consultations that have reached the stage of determining the skull density ratio (SDR). Of these patients, 177 were found to have an adequate SDR of above 0.40, and 50 had an SDR below 0.40. The clinical decisions for these 50 patients with lower SDRs are detailed in Figure 2. Fifteen of these patients with low SDR ultimately underwent HiFU, with the lowest SDR value being 0.26. Among these 15 patients, 5 (33%) achieved complete tremor resolution following HIFU, while 7 (47%) showed a 50–99% reduction in tremor severity. Two patients (13%) experienced no clinical improvement, and one had missing post-operative outcome data. The mean SDR in this cohort was 0.37 (SD: 0.037), and 60% were female. Post-operative side effects were mostly mild or absent, with 43% reporting no adverse effects and 57% reporting only mild side effects. These findings suggest that even in patients with borderline SDR values, HIFU can provide meaningful symptomatic relief with a favorable safety profile.

A preoperative MRI is obtained, and this is used to determine the coordinates for the target lesion location. On the day of the procedure, the first step is shaving the patient’s head, after which they are brought to an MRI suite. A stereotactic frame is affixed to the patient’s head with local anesthetic and pins. An MRI scan is performed, and the previously determined coordinates are confirmed. A series of test sonications to 40–45°C are then performed – these are sonications that are not powerful enough to create a permanent lesion but create a transient effect and aid in ensuring that the targeting is accurate. After this, the therapeutic sonications are performed to achieve target temperature in the range of 56–60°C, and the patient is assessed for adequate clinical effect as well as side effects, with additional sonications performed as needed based on the assessment of the patient tremor suppression and tolerability. A post-operative MRI image is then obtained and the lesion is visualized. The procedure typically lasts less than 4 hours, and intravenous sedation is typically not needed for most patients. Nonetheless, we prefer to perform the procedure with anesthesiology support, as some patients do benefit from small amounts of sedation during frame placement. During the therapeutic sonications, sedation is not usually provided due to the concern of the effects of sedation on the patient’s tremor. Nonetheless, in selected patients, once the decision has been made to perform the final sonication, we may provide sedation to those who will need high energy sonications, as these can be painful.

Patients are able to return home on the same day. Prior to discharge, the patient and family are instructed of the usual post-procedure course, which in the majority of patients is significant for ataxia which can last for days to weeks, and occasionally months. Other less common side effects include paresthesias in the hand, mouth, lips and or tongue, as well as dysarthria and loss of taste, all of which typically resolve in the early postoperative period. Instructions regarding resumption of anticoagulation are also given at discharge time.

## Methods

We searched the literature as of April 2025 using PubMed to identify relevant articles published between 2013 and 2025. Search terms included “high intensity focused ultrasound” AND “essential tremor” as well as “high intensity focused ultrasound” AND “Parkinson’s disease”. 116 articles on HiFU and ET were identified, and 63 articles on HiFU and PD were identified, for a total of 179 articles.

For ET, articles that reported unique results of the HiFU procedure in 5 or more patients were included in our review. Only articles that quantified tremor improvement using the Clinical Rating Scale for Tremor (CRST) hand tremor score were included in the review. These parameters yielded a total of 10 articles on HiFU in ET. For PD, articles that measured efficacy of the HiFU procedure using the motor aspect of the UPDRS (ie, the UPDRS part III) were selected, yielding 7 articles. For both ET and PD, we reviewed data on adverse events from these articles, excluding articles that did not provide data on whether adverse events persisted or resolved.

Additionally, we searched the literature as of April 2025 using PubMed using the search terms “focused ultrasound” AND “bilateral” AND “essential tremor” which yielded 51 articles. From these we selected articles reporting data on 5 or more patients who underwent bilateral HiFU treatment for ET, which yielded 4 articles.

Finally, we reviewed the patients who presented to our center for both HiFU and DBS consultations, and the distance traveled by these patients. We also reviewed the 49 patients who presented for HiFU consultation but were found to have a low SDR, and reported whether these patients ultimately underwent HiFU, DBS, or were managed medically.

## Results/Discussion

### Efficacy of HiFU: Essential Tremor

HiFU was approved for the management of ET in the United States in 2016 following the positive results of a randomized trial published by Elias et. al in 2016 [4]. In this study, patients who had failed two medication trials for ET were randomized in a 3:1 ratio to undergo either unilateral ViM HiFU, or a sham procedure. 56 patients were included in the initial treatment arm, and the study demonstrated a reduction in tremor of almost 40% in this group. Since that time, more than 10,000 cases of HiFU have been performed worldwide at the time of this report. At the present time, follow-up data up to 5 years post-HiFU are available.

We reviewed 10 articles published between 2013 and 2023 on the efficacy of ViM HiFU for the treatment of ET [4,5,6,7,8,9,10,11,12,13]. The majority of the data available consist of prospective studies with the number of participants ranging from 8 to 76. The only randomized trial available for review is the initial trial by Elias et. al in 2016, mentioned above. Supplementary table 1 reviews the demographic data of the patients included in these articles.

**Figure 3:**
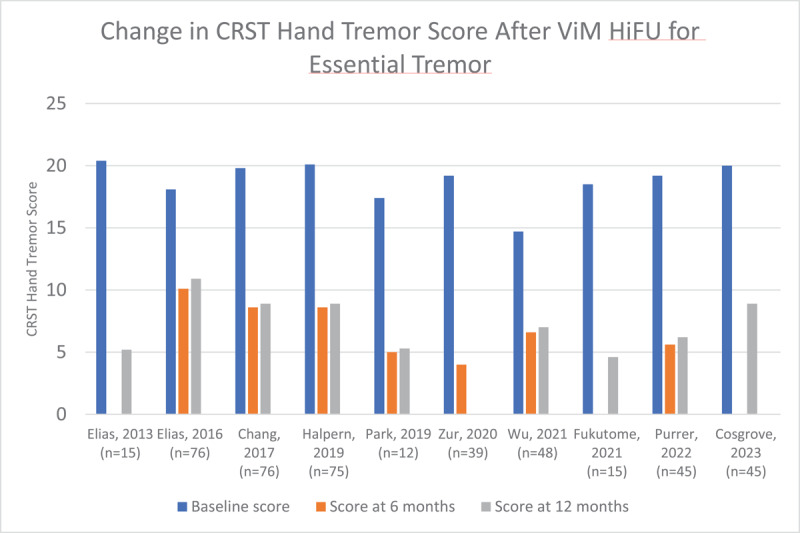
Change in CRST hand tremor score after ViM HiFU for Essential Tremor.

**Figure 4:**
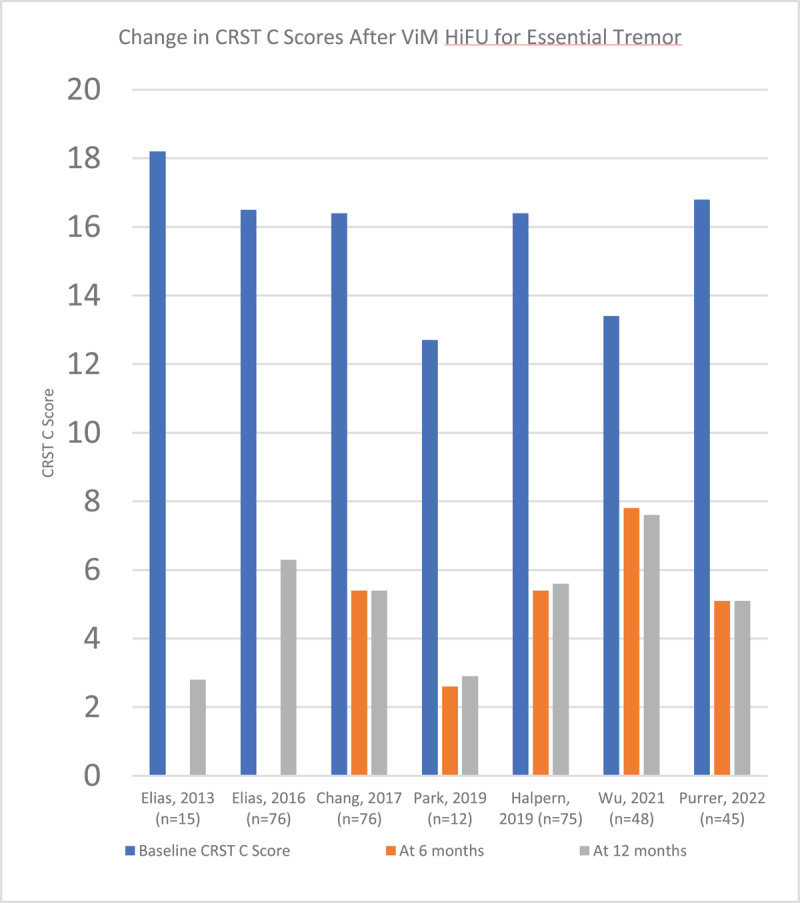
Change in CRST C scores after ViM HiFU for Essential Tremor.

**Figure 5:**
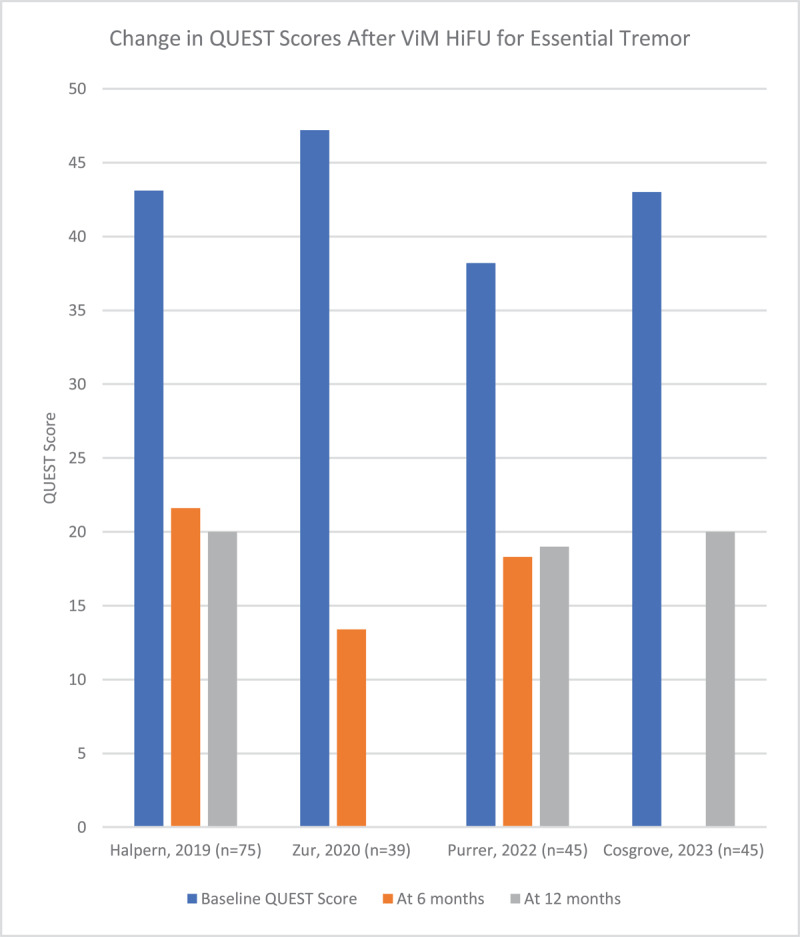
Change in CRST C and QUEST scores after ViM HiFU for Essential Tremor.

Tremor is quantified in these studies using the Clinical Rating Scale for Tremor (CRST). The CRST consists of Part A (global tremor severity), Part B (tremor of the upper extremities during activity), and Part C (functional disability). In the articles selected, the CRST hand tremor score is used, which is a modified score that includes aspects of parts A and B of the CRST, with a maximum score of 32. Figure 3 demonstrates the reduction in CRST hand tremor score after HiFU in the articles that were reviewed. Detailed data including the number of participants in each study and the CRST scores are available in supplementary table 2. Scores focused on disability levels were also tracked in most publications, and the majority used either Part C of the CRST, or the Quality of Life in ET Questionnaire (QUEST). This is illustrated in Figure 4 and Figure 5.

The articles that were reviewed demonstrate a reliable reduction of tremor score post-procedure. Across all studies, the mean percent reduction in hand tremor score from baseline to 6 months was 82.97%, and from baseline to 12 months was 81.51%. Additionally, these improvements were overall reliably sustained at the 12-month mark, with relatively minor increases in hand tremor score noted between 6 months and 12 months. Several studies allowed for longer follow-up ranging between 2 to 5 years, again demonstrating sustained benefit with minor increases in hand tremor score over time. Cosgrove et. al [13] published the most robust 5 year follow-up data, with very minimal increase in hand tremor score at 5 years post-procedure compared to one year post-procedure.

Importantly, these findings did also translate to improvements in functional abilities as reflected by the change in CRST Part C scores, which ranged from a reduction of 43% to 85% at the 12-month mark post-procedure, and improvements in the QUEST score ranging from 53% to 66% at the 12-month mark. Failure of HiFU to produce improvement in tremor was infrequent, and there were few reports of patients choosing to pursue DBS after HiFU due to inadequate tremor control. At our center, of 124 unique patients treated with HiFU for ET, only 1 had to have the procedure repeated for tremor recurrence. In summary, HiFU reliably reduces ET scores in a sustained fashion over the course of several years post-procedure, and this has consistently translated into a reduction in disability due to tremor.

### Bilateral VIM HiFU for Essential Tremor

While bilateral DBS treatments have been performed for 30 years, VIM HiFU was initially approved only as a unilateral procedure due to significant concerns about the effects of bilateral thalamic lesioning, including ataxia and dysarthria. It should be remembered, historically, that the emergence of DBS was due, in large part, because it proved a safer alternative to bilateral thalamotomies which were associated with a high incidence of dysarthria and imbalance. However, as highlighted by Alshaikh and Fishman [14], these concerns stemmed from a number of studies that varied greatly in the method by which the thalamotomy was performed. Many of these studies are over 50 years old, and surgical techniques have significantly advanced since that time. Additionally, the studies demonstrating a high degree of adverse events are those focused on PD – the smaller group of studies focused on ET note a much lower rate of side effects [14]. The ability of HIFU to create a smaller, more controlled lesion offered the hopeful possibility that bilateral ultrasound-lesions would not have the same deleterious effect on speech and balance. The first reported case of bilateral VIM HIFU was reported in 2020 by Ito et. al and provided encouragement to additional studies exploring both the efficacy and tolerability [15].

Since this study, several additional publications have corroborated the effects of bilateral VIM HiFU in ET [16,17,18,19]. This included 4 prospective studies with the number of participants ranging from 5 to 11. A total of 35 patients were included in these studies. The time from the initial lesioning procedure to contralateral lesioning varied from 5 months to one year. Significant improvement in tremor was seen in all patients.

New adverse events that did not resolve after the second procedure included one patient with grade 1 motor neglect, two patients with mild dysarthria or dysesthesia of the tongue, 2 patients with dysphagia, 1 patient with dysgeusia, and 6 patients with hypoesthesia/paresthesia of the face or hand. Any change in gait was reported in a total of 17 out of the 35 patients. These gait changes resolved in 13 of the 17 patients, and data on whether the gait changes resolved in the remaining 4 patients were not available. From this brief review, the side effect profile of the second procedure does not appear markedly different from that of the first side. Bilateral VIM HiFU was approved in December 2022, with a requirement of a 9 month window between each procedure, and we expect that as more data becoms available, the adverse event profile can be assessed more effectively. At our center, we have performed a total of 12 bilateral ViM HiFU procedures, 11 for ET, and 1 for PD. Of these 12 patients, follow up data is available on 10. 100% of these 10 patients reported a 50–99% improvement in tremor on the second side. The side effect profile was similar to that seen with unilateral HiFU, and new unique side effects were not observed. It is worth noting that dysphagia has been described as one of the few side effects to emerge with some latency after bilateral HIFU. At our center we had one patient develop mild but persistent dysphagia to liquids starting three months after the second side. A swallow study was performed, which did not show anu penetration or aspiration. On follow up 1.5 years after the second HiFU procedure was performed, the patient was still experiencing mild dysphagia symptoms, but was much improved.

### Efficacy of HiFU: For Parkinson’s Disease

As was the case with DBS, HiFU was initially approved for use in ET, and its use was then widened to include tremor in PD. DBS initially focused on the ViM as a target, but the focus has now shifted more to the STN and GPI as these targets can effectively address motor symptoms including bradykinesia and rigidity in addition to tremor. DBS remains a highly effective option for tremor control in PD, and in cases where tremor predominates and minimal other motor symptoms are present, ViM HiFU remains a viable consideration for tremor control. In December 2018, HiFU was approved in the United States for treatment of tremor predominant PD. After this, as with DBS, attention turned to targeting the GPi and STN with HiFU, and in 2021 HiFU was approved for treatment of mobility, rigidity and dyskinesias in PD targeting the GPi.

**Figure 6:**
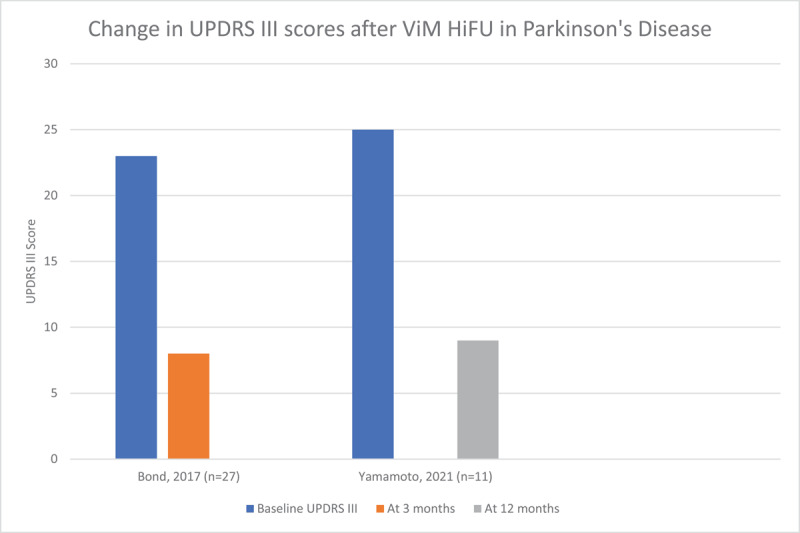
Change in UPDRS part III scores after HiFU in studies targeting the ViM in Parkinson’s disease.

**Figure 7:**
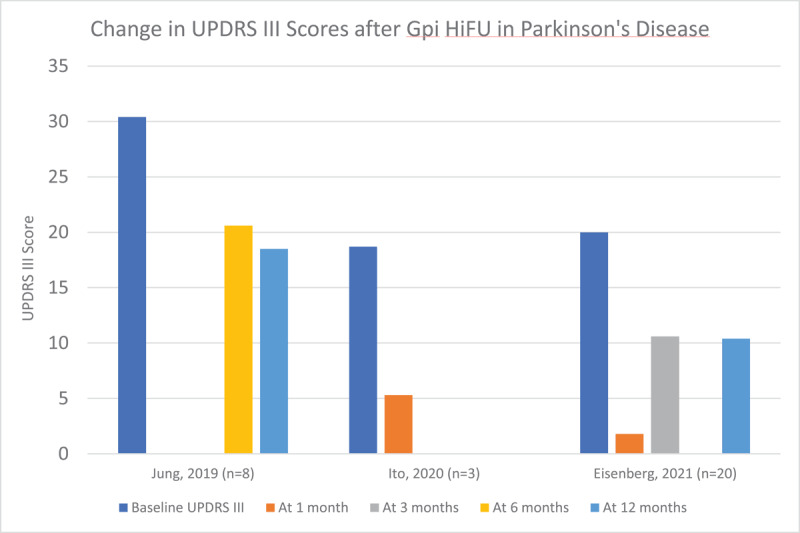
Change in UPDRS part III scores after HiFU in studies targeting the GPi in Parkinson’s disease.

**Figure 8:**
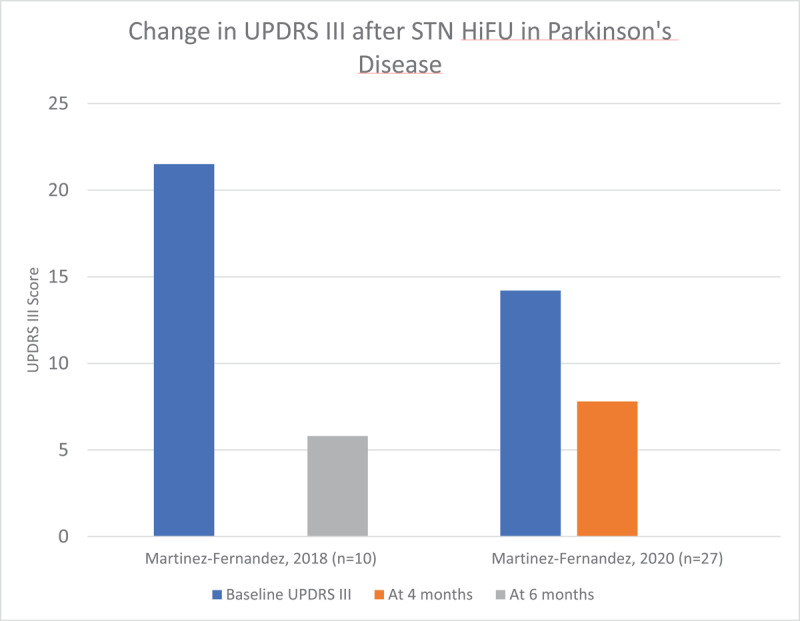
Change in UPDRS part III scores after HiFU in studies targeting the STN in Parkinson’s disease.

**Figure 9:**
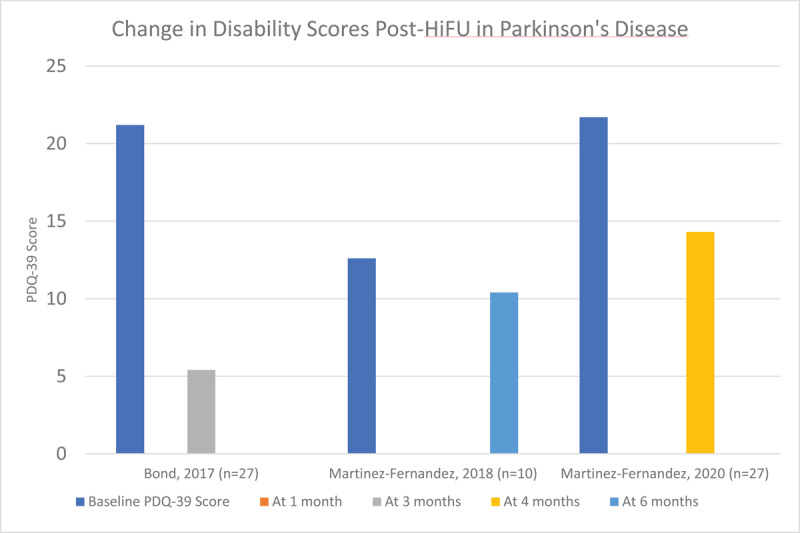
Change in Disability Scores Post-HiFU in Parkinson’s disease, assessed by PDQ-39.

We reviewed 7 articles that reported the results of unilateral HiFU procedures –2 involving the ViM with a total of 40 patients [20,21], 3 involving the GPi with a total of 31 patients [22,23,24], and 2 involving the STN with a total of 37 patients [25,26]. Demographic details of these patients and study data are included in supplementary table 3. Efficacy of the procedure was measured using the motor aspect of the UPDRS (UPDRS part III). Of note, certain studies demonstrated a significant improvement in UPDRS III score in the sham groups, and strong, long-lasting placebo effects have been previously described in patients with PD. Figures 6, 7 and 8 demonstrate the improvement in UPDRS part III score in each cohort. Quality of life was only measured in four of these studies using the PDQ-39 scoring system, and is reported in Figure 9. Three of the four studies demonstrated significant improvement in the PDQ-39 and, as above, one study demonstrated significant placebo effect in the sham arm.

There is a dearth of data on HiFU in PD compared to ET at this point in time. Several authors have commented on a higher degree of tremor recurrence in PD patients compared with ET patients after ViM HiFU [27], with some patients going on to subsequently pursue DBS. Peters et. al [27] commented on the lack of sustained tremor control noted in their Parkinson’s patients after ViM HiFU, which correlated with increasingly high disability scores as patients were followed. It was additionally noted that this was significantly less positive than the results noted in the ET patients. However, other articles do demonstrate effective tremor control with ViM HiFU, and further investigation is needed in this area [19,20,21]. Tremor control has also been reported following STN HIFU but, as will be discussed further below, there are also concerns about the higher rate of adverse events with this target [26].

Additionally, Gallay et. al published the results of 10 patients who had undergone focused ultrasound lesioning of the bilateral pallidothalamic tract (PTT) [28]. The time between the first and second procedure was rarely below one year. At one year follow-up, the mean UPDRS tremor score in the on-medication state was reduced by 91% in these patients, and rigidity, bradykinesia, dystonia, dyskinesias and sleep disorders improved as well. Gait and postural issues remained unchanged, while speech difficulties were increased by 58%. Following this, a larger trial of bilateral PTT lesioning was recently concluded, the results of which are pending at this time. The effects of bilateral lesioning in PD, especially the effects on gait and speech, remain a concern, and will be further assessed in these additional trials.

### Overall Safety of HiFU

Understanding the potential side effects of HiFU is a crucial aspect of selecting and educating candidates for the procedure. The procedure involves deliberately, and ideally permanently, damaging a focused area of the brain by heating tissue to a temperature of approximately 60°C. This invariably leads to a penumbra of tissue that is heated to a lesser degree, with the result that neighboring regions and connections often experience transient dysfunction before recovering. It is thus extremely common for patients to experience transient side effects in the days following the procedure due to indirect heating or post-lesion edema. The decision as to when to conclude the procedure i.e. how many sonications and what temperatures are needed to obtain permanent symptomatic relief while minimizing side effects remains a difficult one, with little objective data available to guide the practitioner. In our experience, we have seen recurrence despite complete tremor relief at appropriate sonication temperature. Our preference is to perform a second sonication slightly dorsal and anterior to a successful sonication at the initial target during the procedure. This is in the hope that a more extensive lesion will correlate with optimal long-term benefit.

### Safety of ViM HiFU

With lesioning of the ViM thalamus, the VC thalamus, a somatosensory nucleus located just posterior to the ViM, may be affected. This can result in contralateral hemi-sensory symptoms such as numbness in the hand, face and tongue, and can also cause hemi-aguesia. Additionally, the internal capsule lies just lateral to the VIM, and when affected the patient may note post-procedure weakness. Effects on the adjacent thalamo-cerebellar connections can also result in imbalance, clumsiness and dysmetria. Together, these sometimes individually subtle side effects can collectively lead to significant gait impairment and heightened fall risk, particularly in patients who have a baseline degree of imbalance – as is not uncommon in both advanced ET and PD. Often patients will not have overt weakness or ataxia on formal testing but will nevertheless report clumsiness with particular tasks such as typing or playing an instrument that may be picked up on exam when looking more carefully at specific activities.

**Figure 10:**
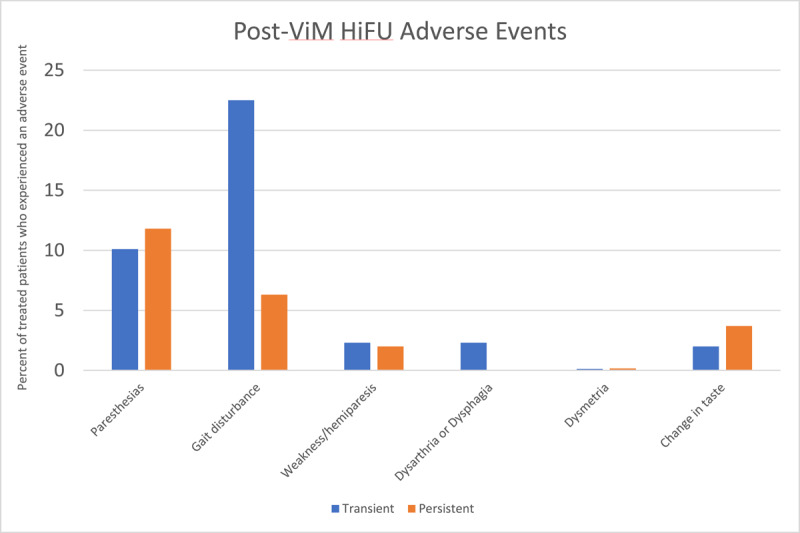
Adverse events after ViM HiFU in Essential Tremor and Parkinson’s Disease.

Figure 10 summarizes the number of patients who developed various side effects after ViM HiFU for ET or PD, based on a pool of 410 patients from the studies that were reviewed. Note that the length of follow up time and extent of information available regarding adverse events was variable in these studies. Additionally, the large cohort of patients studied in Krishna et. al in 2019 was excluded as data on the resolution of adverse events were not available. The most commonly seen adverse events were gait issues (36.8%), paresthesias of the face (13.4%) or of the hand (15.9%), taste disturbances (10.9%), dysmetria (5.1%) and weakness (6.1%). These were also the side effects that were most likely to not fully resolve. Of the 410 patients in this group, 88 patients (21.5%) were persistently experiencing one of these side effects at the most recent follow-up.

### Safety of GPi HiFU

**Figure 11:**
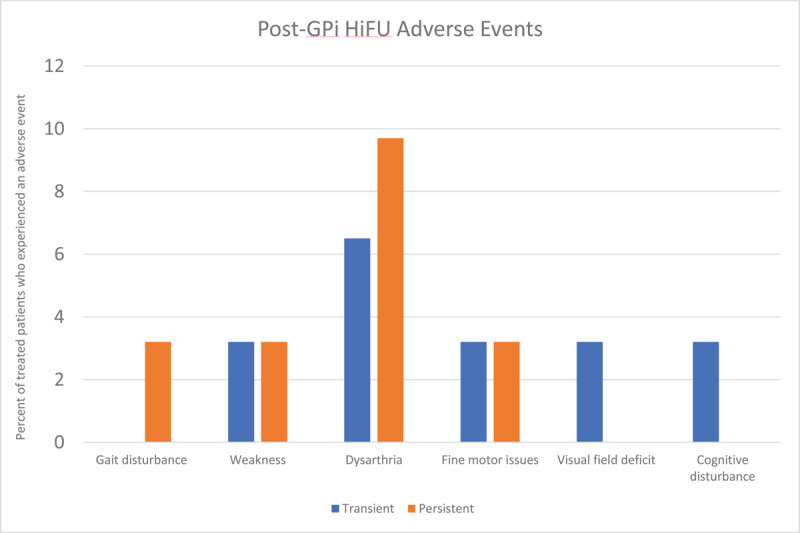
Adverse events after GPi HiFU in Parkinson’s disease.

As with the ViM, the GPi is in close proximity to the internal capsule, leading to the possibility of post-procedural weakness or dysarthria. Proximity to the optic tract can also cause visual field deficits though this has, to our knowledge, not been reported as a lasting side effect. Figure 11 summarizes the number of patients who developed various side effects after GPi HiFU for ET or PD, based on a pool of 95 patients. This is a notably smaller pool of patients in comparison to the data available on adverse events in patients after ViM HiFU. The most commonly seen adverse events were dysarthria or dysphagia (7.4%), gait issues (3.2%), weakness (3.2%), and taste disturbances (2.1%). Of the 95 patients in this group, 6 patients (6.3%) were persistently experiencing one of these side effects at the most recent follow-up. Paresthesias and dysmetria were not seen in the reviewed cases, and would be less likely to occur given the location of the GPi.

### Safety of STN HiFU

The STN is a small structure that is in close proximity to the corticospinal tract, which lies just anterolateral, leading to the possibility of post-procedural weakness or dysarthria. It is also dorsal to the third cranial nerve, which can lead to oculomotor deficits, and lies anterolateral to the medial lemniscus, which can cause paresthesias. Of note, the STN is also a much smaller target than the GPi, which initially led to concerns about whether patients undergoing lesioning of the STN would develop more side effects. Additionally, as lesions of the STN can lead to hemiballismus, there was concern that HiFU of the STN could produce a similar issue. Lastly, as has been noted with DBS, the medial aspect of the STN has significant limbic connections with the potential of inducing mood changes if impacted including mania, impulsivity and depression.

**Figure 12:**
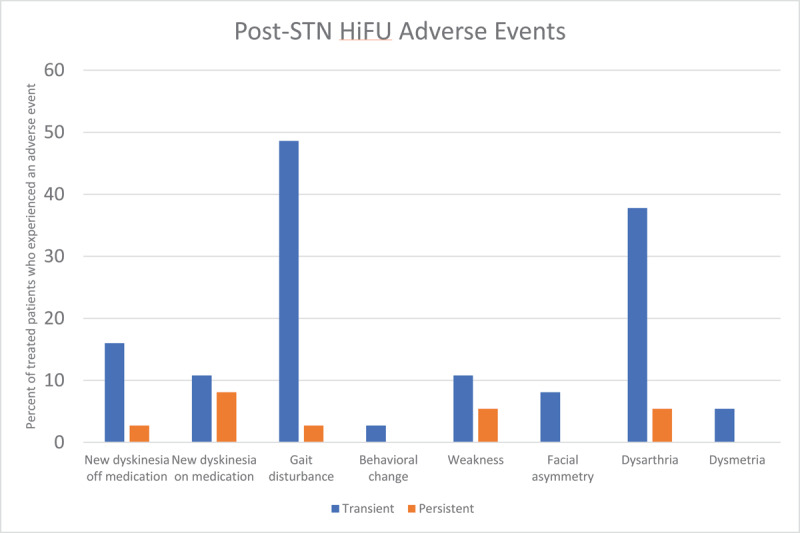
Adverse events after STN HiFU in Parkinson’s disease.

Figure 12 summarizes the number of patients who developed various side effects after STN HiFU for ET or PD, based on a pool of only 37 patients. The most commonly seen adverse events were gait issues (51.4%), dysarthria or dysphagia (40.5%), hemiparesis/weakness (24.3%), new dyskinesias (18.9%), disinhibition/impulsivity (5.4%), and dysmetria (5.4%). Of the 37 patients in this group, 7 patients (18.9%) were persistently experiencing one of these side effects at the most recent follow-up. Chorea/ballism were seen in 6 patients (16.2%), but resolved in all cases. The presence of behavioral changes (disinhibition/impulsivity) and new dyskinesias was relatively unique to this cohort, when compared to the ViM and GPi groups, and the relatively higher prevalence of most side effects in the STN group is notable. However, the STN cohort is a much smaller group of patients than the GPi, and especially the ViM groups, and this highlights a need for more data on adverse events in STN lesioning.

Overall, the risk profile of HiFU, at least for lasting side effects, is quite low which has further increased interest on the part of patients. However, because HiFU has been presented as “non-invasive”, there is often a lack of cognizance as to the likelihood of short-term side effects and the possibility of long-term complications. Therefore, counseling patients about these potential side effects and setting realistic expectations is a crucial element of the pre-procedural education process, with particular attention required in regards to immediate post-procedure fall risk. For a patient whose job or hobbies require exquisite fine motor skills, such as a musician or artist, the possibility of transient or lasting clumsiness and dexterity issues, even if subtle, should be weighed against the benefits of tremor reduction. At our center we have been repeatedly struck by the degree of surprise and discontent among some patients (and their caregivers) who experience short term side effects that were clearly discussed beforehand but somehow not anticipated. Our sense is that despite a concerted effort to review the likelihood of transient side effects, many patients still view it, perhaps subconsciously, as a no-risk alternative to surgery.

Small studies have suggested that patients with peripheral neuropathy and orthopedic issues are more likely to develop significant gait issues post-HiFU, and did not find that older age or pre-existing gait aids were associated with gait decline after HiFU [29]. However, this has not been specifically studied in other publications. Given the high prevalence of gait issues in HiFU patients, even if they are temporary, it is important to inform a patient who is dependent upon a cane or walker that they may need to temporarily use a wheelchair after the procedure. As surgical technique becomes more precise with additional experience, the frequency of side effects may potentially wane, however the correct selection of an appropriate candidate remains critical.

### Comparing HiFU and DBS

Multiple publications have compared HiFU and DBS in terms of efficacy, safety, and longevity, particularly in ET, however the number of matched, prospective cohorts remains limited. Furthermore, the thirty-year history of DBS naturally precludes comparable long-term comparisons with HIFU which has been available mostly unilaterally for less than a decade. Giordano et. al [30] reviewed 37 papers on the outcomes of DBS in ET, and 7 studies on the outcomes of HiFU in ET, for a total of 1202 DBS patients and 477 HiFU patients. Of note, the disease duration was significantly shorter for the HiFU group –18.3 vs 27.2 years. The average percentage improvement in terms of tremor severity was 60.1% for the DBS group and 55.6% for the HiFU group. The average percentage improvement in tremor severity for the DBS group was also significantly higher. However, this has to take into account the fact that almost all DBS cases were done bilaterally, and the HiFU cases were all done unilaterally, as bilateral HiFU was not approved at that time. When a subgroup analysis was done to account for this, DBS remained more effective in terms of tremor control –61.2% vs 56.4%. Notably, however, the average percentage improvement in terms of QoL was 52.5% for the DBS group and 61.9% for the HiFU group. A comparison of complications in the two procedures showed that HiFU was more likely to cause gait issues and paresthesias, and these complications were more likely to be permanent than in DBS. However, DBS caused more cognitive deficits.

In contrast, Harary et. al [31] reported on 127 patients with ET who received ViM DBS, and the majority (80 patients) received unilateral stimulation, while 40 patients received bilateral stimulation. 56 patients underwent unilateral HiFU treatment. The HiFU group was older in mean age (70.8 by 64.6) but otherwise the groups were similar in demographics, and baseline tremor score (CRST Part A) was higher in the DBS cohort than the HiFU group (3.1 vs 2.13). Despite this, results were similar in demonstrating that tremor control in DBS was superior to that in HiFU, with 80% vs 68% improvement in tremor score. Quality of life was measured by the QUEST score, with average 37% and 49% improvement in the DBS and HiFU cohorts, respectively. Statistical significance of this difference could not be established as there was no standard deviation available for the DBS cohort. Again, gait imbalance and paresthesias were the most commonly seen adverse events in the HiFU group, though dysmetria and dysphagia/dysgeusia were noted as well. The DBS cohort experienced a lower frequency of gait disturbance, and one patient noted cognitive changes in comparison to the HiFU group, where no cognitive changes were reported.

The above articles focus on ET patients, as the comparative data on DBS vs HiFU for PD remain quite limited. There is no randomized comparative data on STN/GPi lesioning in PD, and little recent data for the ViM. Any comparisons are further limited by the fact that HiFU is not approved for bilateral treatments in PD at this time.

### How to navigate the data and decision-making process

**Figure 13:**
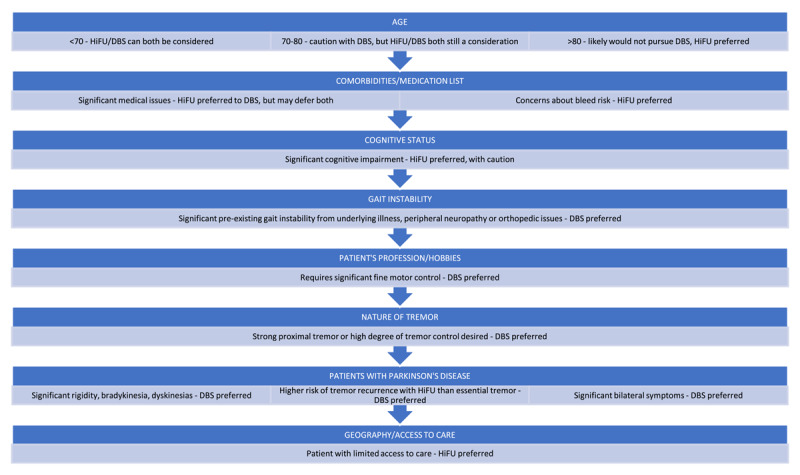
Factors influencing decision-making between HiFU or DBS.

There are many factors to consider when deciding whether DBS or HiFU are the right choice for a patient. Figure 13 reviews these different factors and how they influence decision-making.

One consideration is the patient’s age. A clear age cut-off for DBS has not been established. Many articles suggest that an age below 70 is most favorable, though this largely seems to stem from data suggesting that post-DBS cognitive decline is more likely to occur after age 70, which will be discussed further below. Additional data on mortality in DBS, particularly data looking at patients who died within a number of years after the procedure, has suggested that patients of older ages at the time of operation have a higher mortality risk [32,33,34]. The cause of death is not always clearly available in these cases, and these data could simply reflect the fact that older adults are generally more susceptible to medical issues. There are currently no age restrictions for HiFU, and at our center we have performed the procedure on patients ranging from age 42 to age 97. This includes 34 patients aged 80 and over, and the side effect profile was not noted to be significantly different in these patients.

The next consideration, often linked with age, is the patient’s co-morbidities and medication list which together constitute a good marker of surgical risk. A patient with many co-morbidities may not be appropriate for either procedure, but as HiFU is a less invasive procedure, it may be preferable to DBS. Patients do need to come off of anticoagulation for either procedure, but the risk of bleeding is notably lower for HiFU provided it is not resumed too soon after the procedure (as should be pre-determined in consultation with a cardiologist). No major incidents of bleeding have been noted directly as a result of HiFU though rare cases of post-operative bleeding have been seen following premature resumption of anticoagulation. DBS does carry risk of bleeding, though it remains low. A recent large meta-analysis identified 280 incidents of bleeding in 14,184 lead placements, equating to a bleed rate of 1.97% [35]. Smaller studies have identified bleed rates ranging from 1.12% to 3.7% [36,37]. This should be discussed with patients undergoing DBS, and the risk of coming off of anticoagulation, depending on the reason that the patient is on it, should be considered as well.

The patient’s cognitive status should also be considered early in this process. Significant cognitive impairment generally makes a patient a poor candidate for DBS given the concern about passing electrodes through compromised frontal lobes. Other data also suggest that patients over the age of 70 may be at higher risk for cognitive decline post-DBS [38–39]. Therefore, patients often undergo neuropsychological testing prior to undergoing DBS to assess this risk. By contrast, there is little documented risk of post-procedure confusion or cognitive decline after HiFU, and a recent publication focused specifically on the cognitive safety of HiFU confirmed this [40]. Our center does not routinely perform pre-operative neuropsychological testing for this reason. However, there should nevertheless be heightened cognizance about performing any procedure in patients with significant cognitive impairment particularly given the heightened risk of post-procedure falls should there be prominent confusion. Patients even without evident cognitive impairment at baseline have sometimes anecdotally reported a “brain fog” following the procedure which often improves in the following weeks.

If a patient has gait instability, this should also be a consideration in regards to DBS vs HIFU. As discussed above, many patients experience transient changes in gait after HiFU, which may lead to a patient who already uses a cane or walker needing to temporarily use a wheelchair. This may be considered an unacceptable risk for a patient, even if temporary, and should be discussed in detail. The low, but not zero, chance of persistent gait impairment should be discussed as well. The effect of DBS on gait is complex. Improvement in bradykinesia and rigidity can help with gait but freezing and festination are often less responsive to stimulation as is the ataxia sometimes seen in advanced ET. High levels of stimulation can also sometimes worsen PD and ET gait though in most cases, this can be addressed by adjusting the parameters.

Other side effects that tend to be seen more in HiFU rather than DBS should be discussed as well, such as changes in taste or clumsiness and dysmetria. Each patient has to be considered individually when discussing these side effects- as discussed above, mild clumsiness of the hand can be debilitating for a patient whose job or hobby requires exquisite fine control of the hand. In these cases, DBS may be a better option, if appropriate, as it carries lower risk of post-procedure clumsiness. Many older patients who are simply looking forward to eating and drinking are often not bothered by subtle deficits, even if they are persistent, as long as the basic ADLs are more easily achieved. Side effects such as dysarthria or dysphagia can be seen in either procedure, though in DBS these are more likely to be seen at higher settings, which can then be turned down if desired.

The specific features of the patient’s condition should also be considered when counseling between DBS and HIFU. The nature of the tremor may also help guide decision-making. Although comparative clinical trials are lacking, there is a sense at our center that strong proximal tremors and patients requiring a high degree of tremor control for fine, specific tasks, such as playing an instrument, may do better with DBS given its ability to be continuously adjusted for different circumstances. The advantage, for example, of being able to turn up the settings to perform these fine motor tasks (even if that brings out subtle side effects like dysarthria) is attractive, as after task completion, the settings can then be turned back down. On the other hand, patients with moderate tremors looking for basic improvement in daily tasks may find HIFU equally effective and preferable, especially if older or located far from a specialized center as regards follow-up.

There are additional considerations for the patients who have PD. In those patients who have not only tremor, but also significant rigidity, dyskinesias or bradykinesia, DBS is currently viewed as a more effective option as it can address more features though PTT HIFU has shown promise in this regard and may emerge as a viable alternative in the near future. Additionally, as discussed earlier, the risk of tremor recurrence with HiFU seems higher in the PD population than the ET population. This too may change with time and experience, though it may simply reflect important neurophysiological differences between the two conditions. As HiFU is currently only approved unilaterally for PD, a patient with significant bilateral symptoms may be a better candidate for DBS as well. At this time, we simply do not have comparative long term data, and more investigation is needed on this front. It may be that younger patients with many years of tremor ahead of them may do better with DBS, as it has proven to be efficacious over decades in many, though not all, cases.

It is also of note that DBS can be turned off or reversed if the outcome is not desirable, though removal of electrodes is not without risk, while HiFU creates a permanent lesion that cannot be reversed. DBS could still be pursued after HiFU has been done, while HiFU cannot be done after DBS. Therefore, if HiFU produces inadequate tremor control, it can potentially be repeated in the future for re-emergent tremor, or alternatively DBS can then be pursued, though the risk of adverse events with each additional procedure should be taken into account.

Finally, there are several practical considerations that may influence the treatment choice. Patients need to shave their entire head for HiFU compared with more circumscribed amounts for DBS. HiFU also requires an adequate skull density ratio. DBS, if done bilaterally, will require three procedures – two for the placement of electrodes on each side of the brain, and one for placement of the generator. Post-procedure, the patient will need access to a provider who can remove their sutures, program their device, monitor their battery levels, and charge and arrange for battery replacements when required. If a patient lives in a remote area and is traveling for each of these visits, this can be prohibitively expensive or simply unrealistic. Though HiFU requires imaging pre-procedure, it does not typically require neuropsychiatric testing and is ultimately a single procedure without the need for adjustments, suture removal, or further procedures. This may be a more realistic option for the patient who does not have convenient access to a provider who can manage DBS. We reviewed data on the distance traveled by our patients for DBS and HiFU consultations. The average distance traveled for any consultation was 84.4 miles, with the distance traveled for DBS being only 53.9 miles, while the distance traveled for HiFU was 212.6 miles. This demonstrates how much further the average patient is willing to travel for a one-time treatment with HiFU, in comparison to the multiple visits and routine follow-up required to pursue DBS. Additionally, the stigmata of having something in your body has also proved a major impediment to pursuing DBS. This at least partly explains the relative paucity of DBS for ET at experienced centers, where it remains chiefly utilized for the management of PD.

In summary, a careful and comprehensive review of the patient’s age, co-morbidities, the features of their condition, and their geographic location and access to care, may reveal a clear best option. However, based on the data, an argument can often be made for either procedure without one or the other being clearly superior. In these cases, it is often patient preference and/or a center’s experience and/or biases that leads to the ultimate decision of which to pursue.

## Conclusion

In conclusion, the emergence of HiFU as an effective and generally well-tolerated treatment for movement disorders has allowed for improved quality of life in many patients who might not otherwise undergo treatment. It also, in some cases, allows for a discussion about the relative merits and drawbacks of DBS which patients often have avoided based on preconceived, sometimes inaccurate ideas about risk and what is involved. Identifying appropriate candidates for either intervention remains crucial and involves considering each patient’s circumstances and preferences, potential adverse effects, and practical aspects like access to follow-up and expectations. Data on bilateral HiFU lesioning and emerging targets like the pallidothalamic tract are on the horizon as well and time and experience may alter the landscape still further. This remains a new and evolving field, but one that has generated a lot of enthusiasm from patients. While presently limited to specialized academic centers, it is rapidly expanding and likely to play an increasing prominent role in the management of movement disorders in the decades ahead.

## Additional File

The additional file for this article can be found as follows:

10.5334/tohm.987.s1 Supplementary Data. Tables 1–3.

## References

[1] Chang WS, Jung HH, Zadicario E, Rachmilevitch I, Tlusty T, Vitek S, Chang JW. Factors associated with successful magnetic resonance-guided focused ultrasound treatment: efficiency of acoustic energy delivery through the skull. J Neurosurg. 2016 Feb;124(2):411–6. DOI: 10.3171/2015.3.JNS142592 26361280

[2] Boutet A, Gwun D, Gramer R, Ranjan M, Elias GJB, Tilden D, Huang Y, Li SX, Davidson B, Lu H, Tyrrell P, Jones RM, Fasano A, Hynynen K, Kucharczyk W, Schwartz ML, Lozano AM. The relevance of skull density ratio in selecting candidates for transcranial MR-guided focused ultrasound. J Neurosurg. 2019 May 3;132(6):1785–1791. DOI: 10.3171/2019.2.JNS182571 31051458

[3] Vetkas A, Boutet A, Sarica C, Germann J, Gwun D, Yamamoto K, Jung HH, Alkhotani A, Samuel N, Lang S, Conner CR, Elias GJB, Cheyuo C, Chow C, Santyr B, Iorio-Morin C, Yang AZ, Candeias da Silva C, Fasano A, Kalia SK, Lozano AM. Successful magnetic resonance-guided focused ultrasound treatment of tremor in patients with a skull density ratio of 0.4 or less. J Neurosurg. 2023 Sep 1;140(3):639–647. DOI: 10.3171/2023.6.JNS23171 37657095

[4] Elias WJ, Lipsman N, Ondo WG, Ghanouni P, Kim YG, Lee W, Schwartz M, Hynynen K, Lozano AM, Shah BB, Huss D, Dallapiazza RF, Gwinn R, Witt J, Ro S, Eisenberg HM, Fishman PS, Gandhi D, Halpern CH, Chuang R, Butts Pauly K, Tierney TS, Hayes MT, Cosgrove GR, Yamaguchi T, Abe K, Taira T, Chang JW. A Randomized Trial of Focused Ultrasound Thalamotomy for Essential Tremor. N Engl J Med. 2016 Aug 25;375(8):730–9. DOI: 10.1056/NEJMoa1600159 27557301

[5] Elias WJ, Huss D, Voss T, Loomba J, Khaled M, Zadicario E, et al. A pilot study of focused ultrasound thalamotomy for essential tremor. N Engl J Med. 2013;369(7):640–8. DOI: 10.1056/NEJMoa1300962 23944301

[6] Chang JW, Park CK, Lipsman N, Schwartz ML, Ghanouni P, Henderson JM, Gwinn R, Witt J, Tierney TS, Cosgrove GR, Shah BB, Abe K, Taira T, Lozano AM, Eisenberg HM, Fishman PS, Elias WJ. A prospective trial of magnetic resonance-guided focused ultrasound thalamotomy for essential tremor: Results at the 2-year follow-up. Ann Neurol. 2018 Jan;83(1):107–114. DOI: 10.1002/ana.25126 29265546

[7] Halpern CH, Santini V, Lipsman N, Lozano AM, Schwartz ML, Shah BB, Elias WJ, Cosgrove GR, Hayes MT, McDannold N, Aldrich C, Eisenberg HM, Gandhi D, Taira T, Gwinn R, Ro S, Witt J, Jung NY, Chang JW, Rosenberg J, Ghanouni P. Three-year follow-up of prospective trial of focused ultrasound thalamotomy for essential tremor. Neurology. 2019 Dec 10;93(24):e2284–e2293. DOI: 10.1212/WNL.0000000000008561 31748250

[8] Park YS, Jung NY, Na YC, Chang JW. Four-year follow-up results of magnetic resonance-guided focused ultrasound thalamotomy for essential tremor. Mov Disord. 2019;34(5):727–34. DOI: 10.1002/mds.27637 30759322

[9] Zur G, Lesman-Segev OH, Schlesinger I, Goldsher D, Sinai A, Zaaroor M, Assaf Y, Eran A, Kahn I. Tremor Relief and Structural Integrity after MRI-guided Focused US Thalamotomy in Tremor Disorders. Radiology. 2020 Mar;294(3):676–685. DOI: 10.1148/radiol.2019191624 31909701

[10] Wu P, Lin W, Li KH, Lai HC, Lee MT, Tsai KW, Chiu PY, Chang WC, Wei CY, Taira T. Focused Ultrasound Thalamotomy for the Treatment of Essential Tremor: A 2-Year Outcome Study of Chinese People. Front Aging Neurosci. 2021 Jul 14;13:697029. DOI: 10.3389/fnagi.2021.697029 34335232 PMC8317688

[11] Fukutome K, Kuga Y, Ohnishi H, Hirabayashi H, Nakase H. What factors impact the clinical outcome of magnetic resonance imaging-guided focused ultrasound thalamotomy for essential tremor? J Neurosurg. 2020 May 1;134(5):1618–1623. DOI: 10.3171/2020.2.JNS192814 32357330

[12] Purrer V, Borger V, Pohl E, Upadhyay N, Boecker H, Schmeel C, Pieper CC, Wüllner U. Transcranial high-intensity Magnetic Resonance-guided focused ultrasound (tcMRgFUS) – safety and impacts on tremor severity and quality of life. Parkinsonism Relat Disord. 2022 Jul;100:6–12. DOI: 10.1016/j.parkreldis.2022.05.017 35640415

[13] Cosgrove GR, Lipsman N, Lozano AM, Chang JW, Halpern C, Ghanouni P, Eisenberg H, Fishman P, Taira T, Schwartz ML, McDannold N, Hayes M, Ro S, Shah B, Gwinn R, Santini VE, Hynynen K, Elias WJ. Magnetic resonance imaging-guided focused ultrasound thalamotomy for essential tremor: 5-year follow-up results. J Neurosurg. 2022 Aug 5;138(4):1028–1033. DOI: 10.3171/2022.6.JNS212483 35932269 PMC10193464

[14] Alshaikh J, Fishman PS. Revisiting bilateral thalamotomy for tremor. Clin Neurol Neurosurg. 2017 Jul;158:103–107. DOI: 10.1016/j.clineuro.2017.04.025 28505539

[15] Ito H, Yamamoto K, Fukutake S, Odo T, Yamaguchi T, Taira T. Magnetic resonance imaging-guided focused ultrasound bilateral thalamotomy for essential tremor: A case report. Neurol Clin Neurosci. 2020;8:412–414. DOI: 10.1111/ncn3.12438

[16] Iorio-Morin C, Yamamoto K, Sarica C, Zemmar A, Levesque M, Brisebois S, Germann J, Loh A, Boutet A, Elias GJB, Azevedo P, Adam E, Patel U, Lenis M, Kalia SK, Hodaie M, Fasano A, Lozano AM. Bilateral Focused Ultrasound Thalamotomy for Essential Tremor (BEST-FUS Phase 2 Trial). Mov Disord. 2021 Nov;36(11):2653–2662. DOI: 10.1002/mds.28716 34288097

[17] Fukutome K, Hirabayashi H, Osakada Y, Kuga Y, Ohnishi H. Bilateral Magnetic Resonance Imaging-Guided Focused Ultrasound Thalamotomy for Essential Tremor. Stereotact Funct Neurosurg. 2022;100(1):44–52. DOI: 10.1159/000518662 34515233

[18] Martínez-Fernández R, Mahendran S, Pineda-Pardo JA, Imbach LL, Máñez-Miró JU, Büchele F, Del Álamo M, Rodriguez-Rojas R, Hernández-Fernández F, Werner B, Matarazzo M, Obeso I, Gonzalez-Quarante LH, Deuschl G, Stieglitz L, Baumann CR, Obeso JA. Bilateral staged magnetic resonance-guided focused ultrasound thalamotomy for the treatment of essential tremor: a case series study. J Neurol Neurosurg Psychiatry. 2021 Sep;92(9):927–931. DOI: 10.1136/jnnp-2020-325278 33906933

[19] Scantlebury N, Rohringer CR, Rabin JS, Yunusova Y, Huang Y, Jones RM, Meng Y, Hamani C, McKinlay S, Gopinath G, Sewell IJ, Marzouqah R, McSweeney M, Lam B, Hynynen K, Schwartz ML, Lipsman N, Abrahao A. Safety of Bilateral Staged Magnetic Resonance-Guided Focused Ultrasound Thalamotomy for Essential Tremor. Mov Disord Clin Pract. 2023 Sep 15;10(10):1559–1561. DOI: 10.1002/mdc3.13882 37868927 PMC10585969

[20] Bond AE, Shah BB, Huss DS, Dallapiazza RF, Warren A, Harrison MB, Sperling SA, Wang XQ, Gwinn R, Witt J, Ro S, Elias WJ. Safety and Efficacy of Focused Ultrasound Thalamotomy for Patients With Medication-Refractory, Tremor-Dominant Parkinson Disease: A Randomized Clinical Trial. JAMA Neurol. 2017 Dec 1;74(12):1412–1418. DOI: 10.1001/jamaneurol.2017.3098 29084313 PMC5822192

[21] Yamamoto K, Ito H, Fukutake S, Odo T, Kamei T, Yamaguchi T, Taira T. Focused Ultrasound Thalamotomy for Tremor-dominant Parkinson’s Disease: A Prospective 1-year Follow-up Study. Neurol Med Chir (Tokyo). 2021 Jul 15;61(7):414–421. DOI: 10.2176/nmc.oa.2020-0370 33967176 PMC8280323

[22] Jung NY, Park CK, Kim M, Lee PH, Sohn YH, Chang JW. The efficacy and limits of magnetic resonance-guided focused ultrasound pallidotomy for Parkinson’s disease: a Phase I clinical trial. J Neurosurg. 2018 Aug 1:1–9. DOI: 10.3171/2018.2.JNS172514 30095337

[23] Ito H, Yamamoto K, Fukutake S, Kamei T, Yamaguchi T, Taira T. Two-year follow-up results of magnetic resonance imaging-guided focused ultrasound unilateral pallidotomy for Parkinson’s disease. Neurol Clin Neurosci. 2021;9:73–76. DOI: 10.1111/ncn3.12455

[24] Eisenberg HM, Krishna V, Elias WJ, Cosgrove GR, Gandhi D, Aldrich CE, Fishman PS. MR-guided focused ultrasound pallidotomy for Parkinson’s disease: safety and feasibility. J Neurosurg. 2020 Nov 27;135(3):792–798. DOI: 10.3171/2020.6.JNS192773 33481557

[25] Martínez-Fernández R, Rodríguez-Rojas R, Del Álamo M, Hernández-Fernández F, Pineda-Pardo JA, Dileone M, Alonso-Frech F, Foffani G, Obeso I, Gasca-Salas C, de Luis-Pastor E, Vela L, Obeso JA. Focused ultrasound subthalamotomy in patients with asymmetric Parkinson’s disease: a pilot study. Lancet Neurol. 2018 Jan;17(1):54–63. DOI: 10.1016/S1474-4422(17)30403-9 29203153

[26] Martínez-Fernández R, Máñez-Miró JU, Rodríguez-Rojas R, Del Álamo M, Shah BB, Hernández-Fernández F, Pineda-Pardo JA, Monje MHG, Fernández-Rodríguez B, Sperling SA, Mata-Marín D, Guida P, Alonso-Frech F, Obeso I, Gasca-Salas C, Vela-Desojo L, Elias WJ, Obeso JA. Randomized Trial of Focused Ultrasound Subthalamotomy for Parkinson’s Disease. N Engl J Med. 2020 Dec 24;383(26):2501–2513. DOI: 10.1056/NEJMoa2016311 33369354

[27] Peters J, Maamary J, Kyle K, Olsen N, Jones L, Bolitho S, Barnett Y, Jonker B, Tisch S. Outcomes of Focused Ultrasound Thalamotomy in Tremor Syndromes. Mov Disord. 2024 Jan;39(1):173–182. DOI: 10.1002/mds.29658 37964429

[28] Gallay MN, Moser D, Magara AE, Haufler F, Jeanmonod D. Bilateral MR-Guided Focused Ultrasound Pallidothalamic Tractotomy for Parkinson’s Disease With 1-Year Follow-Up. Front Neurol. 2021 Feb 9;12:601153. DOI: 10.3389/fneur.2021.601153 33633664 PMC7900542

[29] Jackson LM, Kaufmann TJ, Lehman VT, Lee KH, Miller KJ, Hassan A, Klassen BT. Clinical Characteristics of Patients with Gait Instability after MR-Guided Focused Ultrasound Thalamotomy. Tremor Other Hyperkinet Mov (N Y). 2021 Oct 21;11:41. DOI: 10.5334/tohm.643 34721943 PMC8533649

[30] Giordano M, Caccavella VM, Zaed I, Foglia Manzillo L, Montano N, Olivi A, Polli FM. Comparison between deep brain stimulation and magnetic resonance-guided focused ultrasound in the treatment of essential tremor: a systematic review and pooled analysis of functional outcomes. J Neurol Neurosurg Psychiatry. 2020 Dec;91(12):1270–1278. DOI: 10.1136/jnnp-2020-323216 33055140

[31] Harary M, Segar DJ, Hayes MT, Cosgrove GR. Unilateral Thalamic Deep Brain Stimulation Versus Focused Ultrasound Thalamotomy for Essential Tremor. World Neurosurg. 2019 Jun;126:e144–e152. DOI: 10.1016/j.wneu.2019.01.281 30794976

[32] Wider C, Pollo C, Bloch J, Burkhard PR, Vingerhoets FJ. Long-term outcome of 50 consecutive Parkinson’s disease patients treated with subthalamic deep brain stimulation. Parkinsonism Relat Disord. 2008;14(2):114–9. DOI: 10.1016/j.parkreldis.2007.06.012 17822940

[33] Toft M, Lilleeng B, Ramm-Pettersen J, Skogseid IM, Gundersen V, Gerdts R, Pedersen L, Skjelland M, Røste GK, Dietrichs E. Long-term efficacy and mortality in Parkinson’s disease patients treated with subthalamic stimulation. Mov Disord. 2011 Aug 15;26(10):1931–4. DOI: 10.1002/mds.23817 21656853

[34] Hanna JA, Scullen T, Kahn L, Mathkour M, Gouveia EE, Garces, J, Evans LM, Lea G, Houghton DJ, Biro E, Bui CJ, Sulaiman OA, Smith RD. Comparison of elderly and young patient populations treated with deep brain stimulation for Parkinson’s disease: long-term outcomes with up to 7 years of follow-up. Journal of Neurosurgery JNS. 2019;131(3):807–812. DOI: 10.3171/2018.4.JNS171909 30265192

[35] Tiefenbach J, Favi Bocca L, Hogue O, Nero N, Baker KB, Machado AG. Intracranial Bleeding in Deep Brain Stimulation Surgery: A Systematic Review and Meta-Analysis. Stereotact Funct Neurosurg. 2023;101(3):207–216. DOI: 10.1159/000530398 37232022

[36] Topp G, Ghulam-Jelani Z, Chockalingam A, Kumar V, Byraju K, Sukul V, Pilitsis JG. Safety of Deep Brain Stimulation Lead Placement on Patients Requiring Anticlotting Therapies. World Neurosurg. 2021 Jan;145:e320–e325. DOI: 10.1016/j.wneu.2020.10.047 33068799

[37] Wakim AA, Mattar JB, Lambert M, Ponce FA. Perioperative complications of deep brain stimulation among patients with advanced age: a single-institution retrospective analysis. J Neurosurg. 2021 Feb 12;135(5):1421–1428. DOI: 10.3171/2020.8.JNS201283 33578378

[38] Rocha S, Monteiro A, Linhares P, Chamadoira C, Basto MA, Reis C, Sousa C, Lima J, Rosas MJ, Massano J, Vaz R. Long-term mortality analysis in Parkinson’s disease treated with deep brain stimulation. Parkinsons Dis. 2014;2014:717041. DOI: 10.1155/2014/717041 24772365 PMC3960527

[39] Rothlind JC, York MK, Luo P, Carlson K, Marks, WJ Jr, Weaver FM, Stern M, Follett KA, Duda JE, Reda DJ. CSP-468 study group. Predictors of multi-domain cognitive decline following DBS for treatment of Parkinson’s disease. Parkinsonism Relat Disord. 2022 Feb;95:23–27. DOI: 10.1016/j.parkreldis.2021.12.011 34974395

[40] Saporito G, Sucapane P, Bruno F, Catalucci A, Masciocchi C, Pistoia ML, Splendiani A, Ricci A, Di Cesare E, Marini C, Mazza M, Totaro R, Pistoia F. Cognitive safety of focused ultrasound thalamotomy for tremor: 1-year follow-up results of the COGNIFUS part 2 study. Front Neurol. 2024 Jun 17;15:1395282. DOI: 10.3389/fneur.2024.1395282 38952468 PMC11215051

