# Supplementary material for: High Intensity Focused Ultrasound – Longitudinal Data on Efficacy and Safety
Source: Tremor Other Hyperkinet Mov (N Y). 2025 May 5;15:18. doi: 10.5334/tohm.987 (PMC12063574; doi:10.5334/tohm.987)
Supplement: Supplementary Data. — Tables 1–3. [file tohm-15-1-987-s1.pdf]

Supplementary Data

Study	Study design	Number of participants	Mean age	Sex	Mean disease duration in years
Elias et. al, 2013[5]	Prospective	15	66.6±8	67% male	32±21.3 years
Elias et. al, 2016[4]	Randomized trial	Treatment arm: 56<br>Sham/crossover arm: 20	Treatment arm: 70.8±8.7<br>Sham/crossover arm: 71.4 ± 7.3	Treatment arm: 66% male<br>Sham/crossover arm: 75% male	Treatment arm: 28.3±16.4 years<br>Sham/crossover arm: 27.9±14.9
Chang et. al, 2017[6]	Prospective	76	71.0 ± 8.3	68% male	16.8 ± 12.3 years
Halpern et. al, 2019[7]	Prospective	75	71.0 ± 8.3		16.8 ± 12.3
Park et. al, 2019[8]	Prospective	12	61.7 ± 8.1	83% male	17.8 ± 13.03 year s
Zur et. al, 2020 [9]	Prospective	Initial arm: 22<br>Replication arm: 17	Initial arm: 72 years ± 6<br>Replication arm: 73±6	Initial arm: 63% male<br>Replication arm: 59% male	Initial arm: 13± 8<br>Replication arm: 12±9
Wu et. al, 2021 [10]	Prospective	48	59.14 ± 13.5 y	65% male	19.2 ± 13.6
Fukutome et. al, 2021[11]	Prospective	15	62.9 ± 11.3 years	73% male	21.5 ± 14.0
Purrer et. al, 2022[12]	Prospective	37	69.4 ± 12.2	68% male	29.8 ± 16.4
Cosgrove et. al, 2023[13]	Prospective	76	At 5 year follow up, mean age of 75 ± 8.4	75% male	

Table 1: Demographic details for patients included in Figure 3.

Study	Number of participants	Baseline CRST(Clinical Rating Score for Tremor) hand score	Score at 6 months	Score at 12 months	Score at 60 months
Elias et. al, 2013[5]	15	20.4±5.2		5.2±4.8	
Elias et. al, 2016[4]	Treatment arm: 56 patients<br>Crossover patients: 21<br>Sham: 11	Treatment arm: 18.1±4.8<br>Crossover arm: 16.5±4.2<br>Sham arm: 16±4.4	Treatment arm: 10.1±5.3<br>Crossover arm: 8±3.9	Treatment arm: 10.9±4.5<br>Crossover arm: 6.71±4.7	
Chang et. al, 2017[6]	76	19.8 ± 4.9	8.6 ± 4.5	8.9 ± 4.8	
Halpern et. al, 2019[7]	75	20.1 ± 4.7	8.6 ± 4.5	8.9 ± 4.8	
Park et. al, 2019[8]	12	17.4 ± 3.8	5 ± 3.3	5.3 ± 3.4	
Zur et. al, 2020 [9]	Initial arm: 22<br>Replication arm***: 17	Initial arm: 19.2±6.9<br>Replication arm: 18.2±4.3	Initial arm: 4±4		
Wu et. al, 2021 [10]	48	14.7 ± 4.9	6.6 ± 5.3	7.0 ± 5.5	
Fukutome et. al, 2021[11]	15	18.5 ± 5.8		4.6 ± 5.7	
Purrer et. al, 2022[12]	37	19.2 ± 4.2	5.6 ± 4.4	6.2 ± 5.0	
Cosgrove et. al, 2023[13]	40 patients at 5 year follow up	20 ± 4.7		8.9 ± 4.8	9.5 ± 5.4

Table 2: Change in CRST (Clinical Rating Score for Tremor) hand scores post-HiFU.

Study	Study design	Number of participants	Number of participants	Mean age	Sex	Mean disease duration in years
Bond et. al, 2017[20]	Randomized trial	Treatment arm: 20<br>Sham arm: 7	Treatment arm: 20<br>Sham arm: 7	Treatment arm: 68.1<br>Sham arm: 62.4	Treatment arm: 95% male<br>Sham arm: 100% male	Treatment arm: 5.9 (median)<br>Sham arm: 6.7 (median)
Yamamoto et. al, 2021[21]	Prospective	11	11	71.6 ± 6.8	46% male	6.0
Jung et. al, 2019[22]	Prospective	10 patients total*	10	59.8	40% male	10.1
Ito et. al, 2020[23]	Prospective	3	10	67.1±17.5	80% male	
Eisenberg et. al, 2021[24]	Prospective	20	20	56.4±11.3	65% male	9.9±6.4
Martinez-Fernandez et. al, 2018[25]	Prospective	10 patients	10	59.5	60% male	6·3
Martinez-Fernandez et. al, 2020[26]	Randomized trial	Treatment arm: 27<br>Sharm arm: 13	Treatment arm: 27<br>Sham arm: 13	57.1±9.1	65%	6.2±3.0 years

Table 3: Demographic Data for Patients included in Figures 6, 7 and 8.
